# Preoperative ultrasound before sentinel lymph node biopsy in melanoma in the era of neoadjuvant treatment: a systematic review and meta-analysis of diagnostic performance and cost analysis

**DOI:** 10.1016/j.eclinm.2026.103770

**Published:** 2026-01-27

**Authors:** Julia A. van den Broek, Antonius W. Schurink, Brenda Leeneman, Tessa Brabander, Astrid A.M. van der Veldt, Cornelius Verhoef, Dirk J. Grünhagen

**Affiliations:** a Department of Surgical Oncology, Erasmus MC Cancer Institute, Dr. Molewaterplein 40, 3015 GD, Rotterdam, Netherlands; b Erasmus School of Health Policy & Management, Department of Health Technology Assessment, Burgemeester Oudlaan 50, 3062 PA, Rotterdam, Netherlands; c Department of Radiology & Nuclear Medicine, Erasmus Medical Center, Dr. Molewaterplein 40, 3015 GD, Rotterdam, Netherlands; d Department of Medical Oncology, Erasmus MC Cancer Institute, Dr. Molewaterplein 40, 3015 GD, Rotterdam, Netherlands

**Keywords:** Melanoma, Sentinel lymph node biopsy, Ultrasound-guided fine-needle aspiration, Diagnostic accuracy, Cost analysis

## Abstract

**Background:**

Neoadjuvant immunotherapy improves outcomes compared to adjuvant therapy in stage III melanoma and reduces costs when adjuvant therapy is omitted following a major pathological response (MPR). However, adjuvant therapy remains the sole systemic treatment for patients identified as stage III by sentinel lymph node biopsy (SLNB). Detecting nodal metastases prior to this procedure could be beneficial.

**Methods:**

We conducted a systematic review and meta-analysis to determine the accuracy of preoperative ultrasound and fine-needle aspiration cytology (FNAC) and the healthcare costs of implementing this strategy. PubMed, Embase, and Web of Science were searched up to March 21, 2025. Diagnostic cohort studies were included when preoperative ultrasound and/or FNAC were performed in patients with cutaneous melanoma eligible for SLNB, with histopathological confirmation. Studies that lacked individual patient-level diagnostic data were excluded. Two reviewers independently screened and extracted data. The pooled sensitivity and specificity were calculated using bivariate or univariate random-effects models. The associated healthcare costs for each strategy were calculated using the pooled estimates, costs of the procedure, therapies and follow-up.

**Findings:**

Of 1315 records screened, 19 diagnostic studies comprising 7396 patients were included. For ultrasound, pooled sensitivity was 33.6% (95% CI: 23.5–45.5%) and specificity 92.4% (87.3–95.6%). For FNAC, pooled sensitivity and specificity were 92.6% (15.9–99.9%) and 99.1% (96.6–99.8%). Most studies had unclear risk of bias in patient selection and index test domains, while applicability concerns were generally low. Substantial heterogeneity was observed across studies. Ultrasound-FNAC was estimated to detect approximately 31% (8/25.5) of nodal metastases preoperatively. Implementation of this strategy was cost saving across multiple scenarios where adjuvant immunotherapy was omitted following MPR.

**Interpretation:**

Implementation of ultrasound-FNAC prior to sentinel lymph node biopsy enables neoadjuvant immunotherapy and is cost saving, indicating potential value in routine clinical practice.

**Funding:**

None.

**Research in context**

**Evidence before this study**

We searched PubMed, Embase, and Web of Science up to February 2025 for existing systematic reviews and meta-analyses evaluating preoperative ultrasound or ultrasound-guided fine-needle aspiration cytology (FNAC) in patients with cutaneous melanoma eligible for sentinel lymph node biopsy (SLNB), using terms related to melanoma, ultrasound and SLNB. The most recent and comprehensive evidence identified was a 2019 Cochrane systematic review and meta-analysis, which included studies published up to August 2016. This review reported a limited sensitivity of preoperative ultrasound and ultrasound-guided FNAC. As a result, ultrasound-FNAC could not replace SLNB and was not routinely incorporated into melanoma work-up.

**Added value of this study**

This study provides an updated systematic review and meta-analysis of the diagnostic accuracy of ultrasound and ultrasound-guided FNAC in melanoma staging within the current era of neoadjuvant immunotherapy. Although neoadjuvant immunotherapy for stage III melanoma has shown superior outcomes compared with adjuvant therapy, patients undergoing SLNB are only considered for systemic therapy once stage III disease is confirmed, missing the opportunity for neoadjuvant treatment. At the same time, the increasing costs of immunotherapy underscore the importance of evaluating economic implications. To our knowledge, no previous reviews combined diagnostic accuracy estimates with an economic evaluation of preoperative ultrasound-FNAC. In this context, our study shows that ultrasound-FNAC was estimated to detect 31% (8/25.5) of nodal metastases preoperatively, identifying patients eligible for neoadjuvant therapy and consistently resulting in cost savings through the potential omission of adjuvant treatment.

**Implications of all the available evidence**

Preoperative ultrasound-FNAC offers a clinically and economically favorable strategy for staging melanoma in patients eligible for SLNB. This strategy identifies patients who are eligible for neoadjuvant therapy, reduces systemic treatment costs and improves oncologic outcomes.

## Introduction

The incidence of cutaneous melanoma continues to rise, placing an increasing burden on healthcare systems worldwide and underscoring the need for accurate staging and effective treatment strategies.^1^ Sentinel lymph node biopsy (SLNB) has remained a pivotal tool for staging and prognostication in patients with clinically localized melanoma (≥T1b).^2^^,^^3^ Since the introduction of adjuvant therapy, the primary role of SLNB has shifted to identifying patients who are eligible for adjuvant treatment.^4^^,^^5^ More recently, the introduction of neoadjuvant therapy has further transformed the treatment landscape.^5^^,^^6^

This shift toward neoadjuvant treatment has been driven by two trials. The phase 2 SWOG S1801 trial demonstrated that neoadjuvant treatment with pembrolizumab significantly improved event-free survival (EFS) compared to adjuvant pembrolizumab.^7^ Similarly, the phase 3 NADINA trial showed that neoadjuvant ipilimumab plus nivolumab was superior to adjuvant nivolumab in terms of EFS.^8^ According to SWOG S1801, patients were treated with three cycles neoadjuvant and 15 cycles adjuvant treatment. In NADINA, patients received two cycles of ipilimumab plus nivolumab, while adjuvant treatment was guided by pathological response. After neoadjuvant treatment, adjuvant treatment was omitted for patients with a major pathological response (MPR). This de-escalation strategy was feasible in 59% (125/212) of patients. On this basis, neoadjuvant combination therapy is now reimbursed in an increasing number of countries.^5^ As a result, neoadjuvant therapy not only enhances oncological outcomes but also has the potential to reduce overall healthcare costs by reducing the use of adjuvant treatment, as immunotherapy is associated with substantial costs.^9^ Nonetheless, patients with primary melanoma undergoing SLNB are ineligible for neoadjuvant therapy, as nodal involvement is only diagnosed postoperatively.

Preoperative ultrasound has the potential to bridge this diagnostic gap by identifying nodal metastases prior to SLNB. In 2019, a Cochrane meta-analysis reported limited sensitivity (35%, 95% CI: 17.0%–59.4%) but high specificity (94%, 86.1%–97.5%) for ultrasound in detecting lymph node metastases.^10^ This diagnostic performance is insufficient to replace SLNB, as ultrasound cannot reliably exclude nodal involvement and entails additional costs.^11^^,^^12^ As a result, ultrasound has not been widely adopted in daily clinical practice.

The emergence of neoadjuvant treatment options warrants re-appraisal of preoperative ultrasound and fine-needle aspiration cytology (FNAC) prior to SLNB. The aim of this study was to evaluate the diagnostic performance of ultrasound-FNAC through a meta-analysis and to assess the cost implications of implementing preoperative ultrasound-FNAC in patients with melanoma who are eligible for SLNB in the era of neoadjuvant therapy.

## Methods

### Search strategy and selection criteria

This systematic review and meta-analysis followed PRISMA-DTA guidelines.^13^ No review protocol was registered for this study. A search of Medline Ovid, Embase and Web of Science was performed from inception to 21 March, 2025, using terms related to melanoma, SLNB and ultrasound (full search strategy in Supplementary A). Reference lists of included articles were screened manually, and study authors were contacted for clarification or additional data when needed. Eligible studies were full-text, English-language diagnostic cohort studies in patients with cutaneous melanoma eligible for SLNB, using histopathological confirmation by SLNB or lymph node dissection (LND) as the reference standard. Studies were excluded if they involved a wrong setting, index test, study design, insufficient data, and duplicate or related publications. Meta-analyses were performed for preoperative ultrasound and ultrasound-guided FNAC in case of suspicious lymph nodes. Studies reporting on both were included in both analyses if separate 2 × 2 data were available. Two reviewers (J.A., A.W.) independently screened titles and abstracts, followed by full-text review, resolving disagreements by consensus.

### Ethics

Ethical approval and informed consent were not required, as this study involves a systematic review and meta-analysis of published data only.

### Meta-analysis

Data were extracted by one reviewer (J.A.) and checked by a second (A.W.). Variables included study design, sample size, Breslow thickness, index test(s), reference standard, and diagnostic accuracy data at the patient level (true positives (TP), false positives (FP), false negatives (FN), true negatives (TN)). The methodological quality of included studies was assessed using the QUADAS-2 tool, as appropriate.^14^

The primary outcomes of the meta-analysis were sensitivity and specificity. For both ultrasound and FNAC, the sensitivity and specificity with 95% CIs were calculated. A bivariate random-effects meta-analysis (Reitsma^15^) was used to pool sensitivity and specificity, assuming comparable thresholds for test positivity. This approach accounts for the correlation between sensitivity and specificity and incorporates between-study variability.^16^ When bivariate estimation was not feasible or unlikely to yield reliable estimates due to sparse data, multiple zero cells, or limited between-study variability, univariate random-effects logistic regression models were prespecified as an alternative, in accordance with methodological recommendations for diagnostic test accuracy meta-analyses.^17^^,^^18^ These analyses were performed using a generalized linear mixed model with binomial likelihood, logit link, and a random study intercept, allowing zero cells to be accommodated without the use of continuity corrections. Between-study heterogeneity in both bivariate and univariate analyses was represented by the variance of the random effects (τ^2^)^16^ and assessed primarily through visual inspection of forest plots and a summary receiver operating characteristic (SROC) plot. Publication bias was considered qualitatively, based on the comprehensiveness of the search strategy and the characteristics of included studies. Formal tests for funnel plot asymmetry were not conducted, as these are not recommended for diagnostic test accuracy reviews.^16^ Positive and negative likelihood ratios (LR^+^, LR^−^) were calculated, sensitivity analyses were performed and predictive values were stratified by T-stage. All analyses were performed using R (version 4.2.2). There was no funding source for this study.

### Cost analysis

We compared two diagnostic strategies, incorporating multiple scenarios of immunotherapy: (1) proceeding directly to SLNB without prior ultrasound-FNAC (“primary SLNB”), and (2) performing ultrasound-FNAC prior to SLNB (“preoperative ultrasound-FNAC”). The main cost analysis was restricted to patients with T2b or higher melanomas, as patients with T1b and T2a melanomas and a positive sentinel node (SN), classified as stage IIIA, are not consistently considered eligible for adjuvant treatment.^19^^,^^20^ Separate exploratory analyses were conducted for the T1b and T2a subgroups. For each T stage, the proportion of suspicious ultrasound and positive FNAC results were estimated.

For the cost analysis, we simulated the potential medical costs directly related to the treatment pathway. These included diagnostic procedures (ultrasound, FNAC, SLNB), systemic therapy and follow-up.^21^, ^22^, ^23^, ^24^, ^25^ The cost model assumed successful completion of FNAC when indicated. The costs associated with the index node procedure were assumed to be comparable to those of SLNB, given the similar surgical and pathological workflow. All patients were modelled to adhere fully to the relevant clinical trial protocols. The sensitivity and specificity from the meta-analysis of ultrasound and FNAC were used in the model. Data on clinical and melanoma-specific outcomes were obtained from phase III clinical trials and observational data, including administrative registries and published literature (Supplementary B). Healthcare costs were collected in accordance with the Dutch costing manual^22^ and indexed to 2025 price levels using the general consumer price index published by the Statistic Netherlands.^26^

Drug costs for systemic therapy were obtained from the national Dutch drug cost database,^21^ calculated based on flat dosing per cycle. Several scenarios were modelled. For adjuvant treatment, there were three regimens: 18 cycles of pembrolizumab (200 mg), 15 cycles of nivolumab (240 mg), and a 52-week course of dabrafenib (150 mg twice daily) combined with trametinib (2 mg once daily). The two neoadjuvant treatment scenarios included two 3-weekly cycles of ipilimumab (80 mg) plus nivolumab (240 mg), and three 3-weekly cycles of pembrolizumab (200 mg). Following neoadjuvant treatment, patients underwent an index node resection to assess pathological response, which was proven as a reliable surrogate for overall nodal response.^27^ The ipilimumab plus nivolumab scenario, modelled according to the NADINA trial protocol,^8^ was designated as the primary scenario: patients who did not achieve MPR (41%, 87/212) received adjuvant therapy: either 11 cycles of nivolumab (480 mg) or, for BRAF-mutated cases (assumed 46% based on published data^28^), dabrafenib plus trametinib for 46 weeks. For neoadjuvant pembrolizumab, multiple postoperative treatment scenarios were evaluated, including the standard adjuvant regimen of 15 cycles and an exploratory response-driven approach in which adjuvant therapy was administered based on pathologic response. As the MPR rate for pembrolizumab is not yet well established, the model assumed a range of MPR rates from 10% to 50% to reflect potential clinical outcomes.

### Role of the funding source

There was no funding source for this study.

## Results

**Fig. 1:**
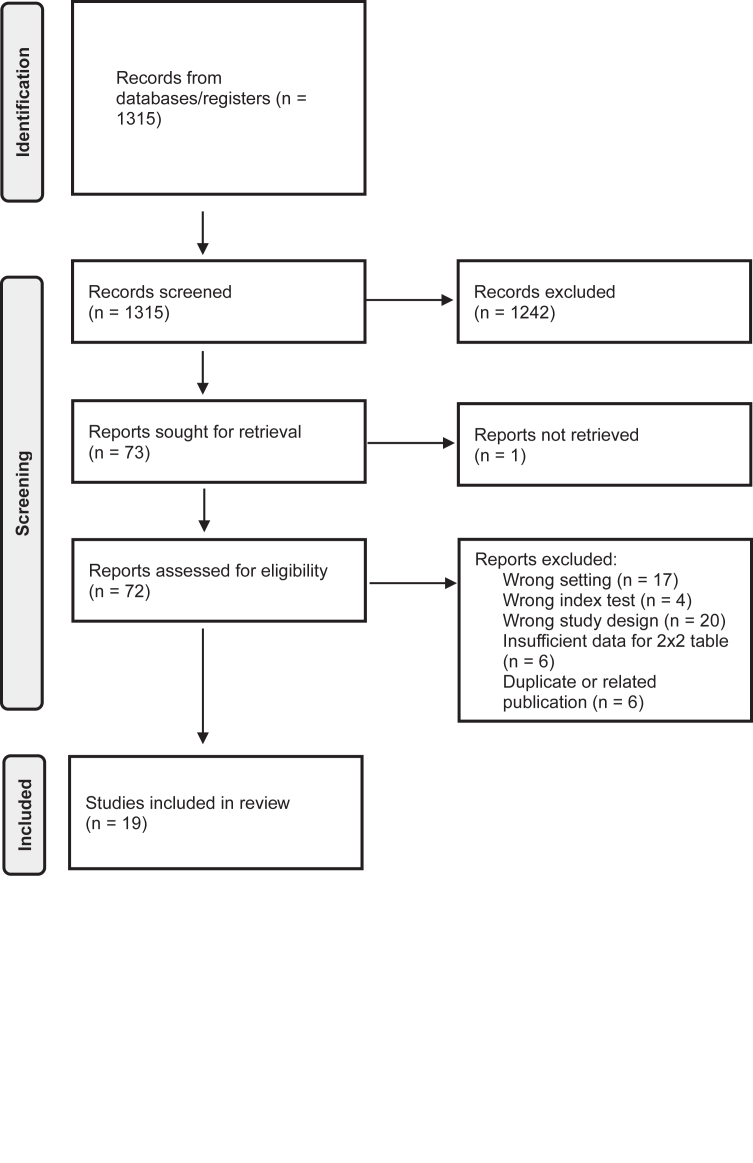
**PRISMA flow chart - search and selection of studies**.

The search yielded 1315 records after deduplication (Fig. 1). 19 diagnostic studies comprising 7396 patients were included. 18 studies analyzed ultrasound, and 5 ultrasound-guided FNAC in patients with suspicious findings on ultrasound; four contributed to both analyses (Table 1).

**Table 1:** Included studies evaluating preoperative ultrasound and/or ultrasound-guided FNAC in patients with melanoma eligible for SLNB.

Author, year	Interventions	Study design	Breslow analysis	Prevalence (+SN/n)	%
Thompson,^29^ 2021	Ultrasound	Retrospective, multicenter	+	548/2859	19%
Prkacin,^30^ 2021	Ultrasound	Unknown		7/101	7%
Stahlie,^31^ 2021	FNAC	Prospective, monocentric		14/23	61%
Riquelme-McLoughlin,^32^ 2019	Ultrasound	Prospective, monocentric	+	250/32	13%
Kwon,^33^ 2019	Ultrasound	Retrospective, monocentric		24/98	24%
Ternov,^34^ 2018	Ultrasound	Prospective, monocentric		20/91	22%
Olmedo,^35^ 2017	Ultrasound	Retrospective, monocentric		102/384	27%
Voit,^36^ 2014	Ultrasound, FNAC	Prospective, monocentric	+	208/1000	21%
Hinz,^37^ 2013	Ultrasound	Retrospective, monocentric		12/20	60%
Marone,^38^ 2012	Ultrasound	Prospective, monocentric		95/623	15%
Chai,^39^ 2012	Ultrasound, FNAC	Retrospective, monocentric		71/325	22%
Pilko,^40^ 2012	Ultrasound	Retrospective, monocentric		115/405	28%
Hinz,^41^ 2011	Ultrasound	Prospective, monocentric		8/81	10%
Sanki,^42^ 2009	Ultrasound	Prospective, monocentric	+	125/716	17%
Kunte,^43^ 2009	Ultrasound	Prospective, monocentric		6/25	24%
Sibon,^44^ 2007	Ultrasound	Prospective, monocentric		35/131	27%
Van Rijk,^45^ 2006	Ultrasound, FNAC	Retrospective, monocentric		37/107	35%
Hocevar,^46^ 2004	Ultrasound, FNAC	Prospective, monocentric		14/57	25%
Hafner,^47^ 2004	Ultrasound	Prospective, monocentric		23/100	23%

Abbreviations: FNAC, fine-needle aspiration cytology; SLNB, sentinel lymph node biopsy; SN, sentinel node; n, number.

Most studies had unclear risk of bias for patient selection due to insufficient reporting on sampling methods; two had high risk from inappropriate exclusions.^29^^,^^30^ Applicability concerns were generally low, though some studies focused only on advanced-stage or acral melanoma.^31^^,^^32^ For the index test, risk of bias was frequently unclear due to lack of blinding and limited detail on sonographic criteria or operator expertise. One study incorporated clinical follow-up in the reference standard, potentially introducing bias. In the flow and timing domain, missing interval data and unexplained exclusions raised concerns about incomplete outcome data and possible information bias.^33^ A summary is presented in Supplementary C.

For ultrasound, pooled sensitivity was 33.6% (95% CI: 23.5–45.5%) and specificity 92.4% (87.3–95.6%) using a bivariate random-effects model. For FNAC, a univariate random-effects logistic regression model was applied, given the limited dataset of five studies with sparse data and multiple zero cells, three of which reported 100% sensitivity and specificity. The pooled sensitivity and specificity for FNAC were 92.6% (95% CI: 15.9–99.9%) and 99.1% (96.6–99.8%), respectively. Corresponding likelihood ratios were 4.42 (LR^+^) and 0.72 (LR^−^) for ultrasound, and 103 (LR^+^) and 0.07 (LR^−^) for FNAC. Sensitivity analyses using a leave-one-out approach showed similar results, supporting the robustness of the estimates.

**Fig. 2:**
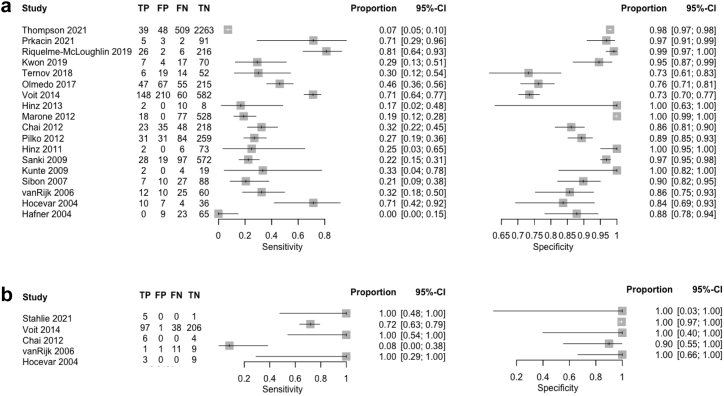
**a: Forest plot ultrasound. b: Forest plot fine-needle aspiration cytology**. Abbreviations: TP, true positive; FP, false positive; FN, false negative; TN, true negative

**Fig. 3:**
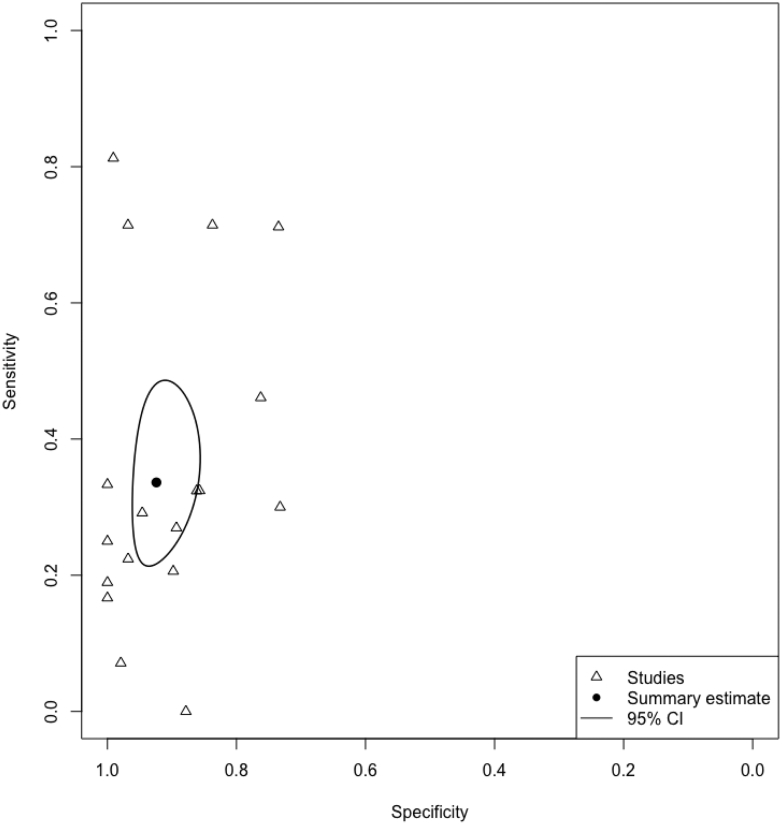
**Summary receiver operating characteristic plot—ultrasound**.

The bivariate random-effects model for ultrasound included between-study variance components for logit-sensitivity (τ^2^ = 0.94) and logit-specificity (τ^2^ = 1.23). In the univariate random-effects logistic regression model for FNAC, variance between studies was apparent for sensitivity (τ^2^ = 11.09), whereas specificity estimates were relatively consistent across studies (τ^2^ = 0). These patterns are illustrated in the forest plots for ultrasound and FNAC and the SROC plot for ultrasound (in Figs. 2 and 3). One study was identified as an outlier in the FNAC forest plot, with a low sensitivity of 8% (1/12).^34^ In this study, 11 of the 20 FNAC-negative cases were found to have nodal metastases on histopathology, indicating a high false-negative rate.

**Fig. 4:**
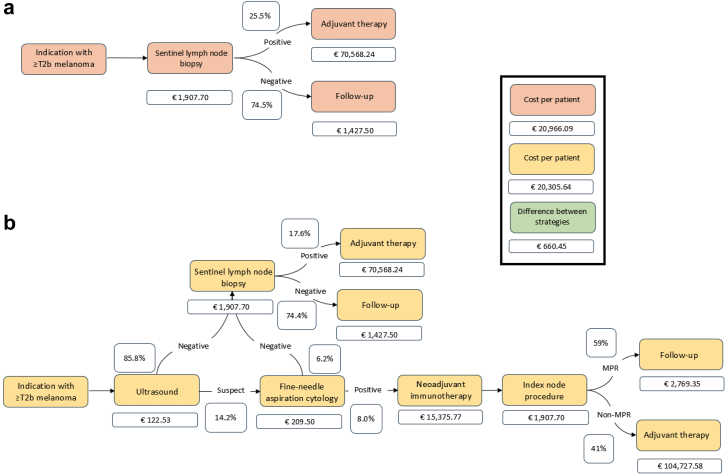
**a: Overview of the two diagnostic strategies–primary sentinel lymph node biopsy strategy. b: Preoperative ultrasound and fine-needle aspiration cytology strategy**.

The two strategies compared in the cost model are illustrated in Fig. 4, which outlines diagnostic and treatment pathways, corresponding probabilities, and stepwise costs. Based on the literature, the nodal metastasis rate among patients with T2b or higher-stage melanoma is approximately 25.5% (range 14.5% for T2b–34.5% for T4).^35^^,^^36^ Using this pre-test probability and the pooled diagnostic accuracy estimates, the model-derived probability of a positive preoperative FNAC result was 8% for all patients. Consequently, ultrasound-guided FNAC was estimated to identify approximately 31% (8/25.5) of nodal metastases prior to SLNB, while the remaining 69% would be detected through SLNB. A detailed stratification by T stage, including predictive values reflecting the post-test probability of nodal involvement, is provided in Supplementary D.

In the primary SLNB strategy, 25.5% of patients had a positive SN and received adjuvant nivolumab, while 74.5% proceeded to routine follow-up following a negative SN. The scenario with adjuvant nivolumab yielded an average per-patient cost of €20,966.09.

The preoperative ultrasound-FNAC strategy alters treatment allocation. The following scenario is based on the regimen used in the NADINA trial. In the model, 8% of all patients were FNAC-positive and received neoadjuvant ipilimumab plus nivolumab. Among them, 59% achieved an MPR and required no further adjuvant treatment. 41% did not achieve MPR and were treated postoperatively with either adjuvant nivolumab (37%) or dabrafenib plus trametinib (63%), according to data from the NADINA trial based on BRAF mutation status.^8^ The remaining 92% of all patients proceeded to SLNB, of whom 17.6% had a positive SN and received adjuvant nivolumab, while 74.4% continued routine follow-up (Fig. 4). This strategy reduced the average per-patient cost to €20,305.64, yielding a cost reduction of €660.45 compared with SLNB followed by adjuvant nivolumab and the neoadjuvant NADINA regimen.

Across all modelled scenarios, the use of neoadjuvant ipilimumab plus nivolumab consistently yielded overall cost savings (Table 2). Most scenarios incorporating neoadjuvant pembrolizumab were also cost saving, particularly when adjuvant pembrolizumab following neoadjuvant pembrolizumab was based on pathological response (Supplementary E).

**Table 2:** Cost analysis comparing primary SLNB versus preoperative ultrasound-FNAC, including scenarios for adjuvant treatment following a positive sentinel node and for patients receiving neoadjuvant ipilimumab–nivolumab with non-MPR in the index node resection.

Adjuvant following a positive SN	Adjuvant following non-MPR after neoadjuvant ipilimumab-nivolumab	Primary SLNB	Pre-operative ultrasound-FNAC	Difference
Nivolumab	Nivolumab	€20,966.09	€18,845.85	€2120.24
Pembrolizumab	Nivolumab	€28,491.20	€24,029.63	€4461.58
Nivolumab	Nivolumab/dabrafenib-trametinib	€20,966.09	€20,305.64	€660.45
Pembrolizumab	Nivolumab/dabrafenib-trametinib	€28.491.20	€25,489.42	€3001.78
Nivolumab/dabrafenib-trametinib	Nivolumab/dabrafenib-trametinib	€30,005.24	€26,532.39	€3472.85
Pembrolizumab/dabrafenib-trametinib	Nivolumab/dabrafenib-trametinib	€34,068.81	€29,331.63	€4767.17

Abbreviations: SN, sentinel node; MPR, major pathological response; SLNB, sentinel lymph node biopsy; FNAC, fine-needle aspiration cytology.

## Discussion

In the meta-analyses of ultrasound and FNAC, pooled sensitivity and specificity of preoperative ultrasound were 33.6% (95% CI: 23.5–45.5%) and 92.4% (87.3–95.6%), respectively, and for ultrasound-guided FNAC 92.6% (95% CI: 15.9–99.9%) and 99.1% (96.6–99.8%). In patients with T2b melanoma or higher-stage eligible for SLNB, preoperative ultrasound-FNAC would detect 31% (8/25.5) of all nodal metastases. The preoperative detection of nodal metastases in clinically node-negative patients enables neoadjuvant treatment with a view to improve outcomes. This approach results in cost savings irrespective of the adjuvant treatment strategy applied.

In 2019, a Cochrane meta-analysis reported a sensitivity of 35% (95% CI: 17.0%–59.4%) and specificity of 94% (86.1%–97.5%) for preoperative ultrasound in patients eligible for SLNB, which is consistent with our pooled estimates.^10^ This is further supported by a meta-analysis by Xing et al., which showed that ultrasound had the highest sensitivity and specificity for detecting lymph node metastases among various imaging modalities (PET/CT and CT) in patients who are eligible for SLNB.^37^ The sensitivity of the FNAC meta-analysis is higher compared to previously reported outcomes (18%, 95% CI: 3.58%–56.5%^10^), likely due to newer studies and methodological differences, such as the use of univariate instead of bivariate analysis. Our analysis further extends previous work by integrating the latest evidence, including ultrasound data from the MSLT-II trial.^38^

Since January 2025, the updated European Society for Medical Oncology (ESMO) guideline recommends ultrasound of the loco-regional lymph nodes as part of the initial workup for patients with T1b melanoma or higher.^11^ This recommendation is based on expert consensus and aims to increase the proportion of patients who are eligible for neoadjuvant rather than adjuvant treatment, as neoadjuvant treatment improves EFS compared to adjuvant therapy.^7^^,^^8^ Neoadjuvant ipilimumab plus nivolumab improved 12-month EFS from 57.2% (99.9% CI: 45.1–72.7) to 83.7% (73.8–94.8), while neoadjuvant pembrolizumab improved 2-year EFS from 49% (95% CI: 41–59) to 72% (64–80). The proposed strategy with preoperative ultrasound-FNAC could result in detection of smaller metastases than those included in the pivotal neoadjuvant trials, which enrolled only patients with macroscopic stage III disease. Whether patients with small nodal metastases derive the same benefit from neoadjuvant treatment is unknown. Accordingly, incorporating ultrasound into the initial diagnostic workup could improve clinical outcomes, as well as contribute to overall cost reduction.

In our analysis, we evaluated both neoadjuvant treatment strategies. Across all modelled scenarios, neoadjuvant treatment with ipilimumab plus nivolumab consistently resulted in cost savings, primarily due to the omission of costly adjuvant therapy in patients achieving MPR. Neoadjuvant treatment with pembrolizumab was also cost-saving in scenarios where adjuvant therapy was omitted following an MPR, an approach that is not yet standard clinical practice.^5^^,^^6^ Omission of adjuvant therapy based on pathological response after neoadjuvant pembrolizumab has not yet been investigated. This approach may become an option pending further validation of its safety and efficacy in future clinical studies.

Preoperative detection of melanoma deposits in lymph nodes by ultrasound-FNAC in clinically node-negative patients imposes a staging dilemma. According to the current AJCC 8th edition staging system, clinically occult stage III disease refers to nodal metastases detected by SLNB, whereas clinically evident stage III disease is defined by clinically or radiologically detected nodal involvement.^3^ This distinction has implications for surgical management of the nodal basin: patients with clinically evident nodal metastases have traditionally been considered candidates for LND. However, for patients with clinically occult stage III disease, LND represents overtreatment. Landmark trials such as DeCOG-SLT and MSLT-II demonstrated that LND following a positive SN has no significant survival benefit, while being associated with substantial morbidity (24%).^39^, ^40^, ^41^ It has been shown that ultrasound-detected SN involvement is not an independent predictor of melanoma-specific survival,^12^ thereby it could be argued that a standard LND is likely unnecessary for a positive SN detected by ultrasound-FNAC. Therefore, LND was not incorporated in our cost analysis. Notably, response assessment following neoadjuvant treatment can be reliably performed through surgical removal of the index lymph node.^27^^,^^42^ In patients who do not achieve MPR, LND may still be considered.

Preoperative ultrasound-FNAC was not modelled for patients with T1b or T2a melanoma, as these melanomas can only be upstaged to IIIA following SLNB.^43^ Current international guidelines either do not routinely recommend adjuvant therapy for stage IIIA disease or limit eligibility to cases with SN metastases exceeding 1 mm or 0.3 mm, respectively.^44^^,^^45^ Scenario analyses (Supplementary Table E) showed that applying this strategy to T1b–T2a melanomas would result in higher overall expenditures, with the eligibility criteria of 1 mm tumor burden for adjuvant immunotherapy. Moreover, diagnostic performance of ultrasound appears to correlate with Breslow thickness. Several studies included in our systematic review reported higher sensitivity in patients with higher T-stage (Supplementary F), further supporting our decision to restrict the cost analysis to patients with T2b melanoma and above.

This study has several limitations. First, the diagnostic accuracy of preoperative ultrasound and FNAC varied considerably across studies, reflecting heterogeneity in locations of lymph node basins and methodological differences. Inter-operator variability is another well-recognized issue and a plausible driver of the heterogeneity observed in our meta-analysis. While some centers employed dedicated sonographers,^29^, ^30^, ^31^^,^^46^^,^^47^ others relied on general radiologists with mixed experience levels,^34^^,^^48^^,^^49^ suggesting the pooled estimate for sensitivity could be applicable to specialized melanoma centers. The impact of operator experience was evident in the study van Rijk et al., which reported a FNAC sensitivity of only 8% (1/12) when seven radiologists with variable expertise were involved,^33^ and in the study Voit et al., where sensitivity declined after transitioning from a single expert to less experienced radiologists.^34^^,^^50^ Second, sex distribution was not consistently reported across included studies,^38^^,^^46^^,^^48^^,^^51^ limiting assessing sex-specific diagnostic performance. Third, the possibility of publication bias cannot be excluded, as studies with negative or inconclusive findings might be underrepresented. Fourth, different statistical approaches for pooling proportions may yield different point estimates.^52^ In line with diagnostic test accuracy guidance, random-effects models were prespecified and applied.^16^^,^^18^ The pooled estimates should be interpreted with caution, with emphasis on the wide confidence intervals and overall uncertainty. Finally, FNAC estimates in the current meta-analysis were based on successfully completed aspiration, although FNAC is not always feasible in clinical practice due to insufficient material, logistical constraints and inaccessible lymph node location.^46^^,^^47^^,^^50^^,^^53^ In our cost analysis, we assumed a 100% FNAC success rate, which may overestimate its efficiency. Given that the cost savings in the least favorable scenario amounted to €660 and that ultrasound with FNAC costs €334, our results suggest that up to 50% of FNAC procedures could fail while maintaining overall cost savings. These findings underscore the importance of involving radiologists with specific expertise in ultrasound-guided FNAC of lymph nodes to optimize diagnostic accuracy.

Regarding the cost analysis, only direct medical costs were included, excluding indirect and societal costs. The cost-saving potential may differ under broader health economic perspectives. Moreover, the cost analysis was deterministic and did not incorporate parameter uncertainty, and some cost estimates may therefore be subject to error. Our findings are based on Dutch healthcare pricing, which may limit generalizability to other countries as unit prices for diagnostic tests, surgical procedures, and oncologic therapies vary across healthcare systems. Although the absolute magnitude may differ, the high price of immunotherapy is a consistent global driver of melanoma care expenditures, suggesting that the cost savings associated with preoperative ultrasound-FNAC are expected to demonstrate comparable effects across countries.

This cost analysis reflects current clinical practice, in which all patients were modelled to undergo and complete (neo)adjuvant therapy. However, the therapeutic and economic context is likely to shift over time. This is driven by emerging clinical data, the expiration of anti–PD-1 patent protection, and advancements in risk stratification such as gene-expression profiling and biomarker-based selection for systemic therapy.^54^ These developments may alter treatment pathways and associated costs. As such, a reassessment of cost-effectiveness will be warranted. Additionally, when data from the NADINA^8^ and SWOG S1801^7^ trials mature, patient-level cost-effectiveness analyses should be conducted, incorporating variables such as immune-related adverse events (irAEs), which were not included in the present study and could possibly influence the net savings. Notably, the incidence of grade 3 or higher irAEs with ipilimumab plus nivolumab is 29.7% (63/212), compared with 14.7% (25/170) for adjuvant nivolumab,^8^ underscoring the importance of integrating toxicity into future evaluations.

In the evolving landscape of melanoma care with the introduction of neoadjuvant therapy, re-evaluation of the current staging workup is warranted. Among patients with T2b or higher melanoma who were eligible for SLNB, preoperative ultrasound-FNAC was estimated to detect 31% (8/25.5) of all nodal metastases, corresponding to an estimated 8% of the overall patient population being identified as candidates for neoadjuvant therapy. Despite this modest detection rate, the approach remained cost-saving, primarily due to the potential reduction in the use of expensive systemic therapies. Across multiple cost scenarios, preoperative ultrasound-FNAC consistently resulted in cost savings. Further evaluation of this treatment trajectory is warranted to validate its impact in real-world practice. The findings from this study highlight the potential value of incorporating preoperative ultrasound-FNAC into the initial staging of patients with a T2b or higher melanoma, particularly in specialized centers.

## Contributors

DJG conceived and supervised the study. JAvB and AWS developed the methods, acquired the data, performed the formal analysis, and prepared the figures. BL and TB contributed to methodology and validated the findings. AAMvdV, CV, and DJG provided supervision and contributed to conceptualization. JAvB and AWS wrote the original draft of the manuscript. All authors interpreted the results and contributed to reviewing and editing the article. JAvB and AWS accessed and verified all underlying data. All authors had full access to all the data in the study, and had final responsibility for the decision to submit for publication. All authors read and approved the final version of the manuscript.

## Data sharing statement

All data used in this meta-analysis were extracted from previously published studies and are fully presented in the manuscript. The statistical code used for the analyses is available from the corresponding author upon reasonable request.

## Declaration of interests

AAMV provided consultancy for BMS, MSD, Sanofi, Merck, Pierre Fabre, Ipsen, Roche, Novartis, Eisai and Pfizer (all fees were paid to the institution). She has also received travel support from Ipsen. AAMV and DJG are members of the Dutch national melanoma guideline committee. All other authors have declared no conflicts of interest.
