# Supplementary material for: Preoperative ultrasound before sentinel lymph node biopsy in melanoma in the era of neoadjuvant treatment: a systematic review and meta-analysis of diagnostic performance and cost analysis
Source: eClinicalMedicine. 2026 Jan 27;92:103770. doi: 10.1016/j.eclinm.2026.103770 (PMC12865617; doi:10.1016/j.eclinm.2026.103770)
Supplement: Supplementary Material [file mmc1.pdf]

SUPPLEMENTARY MATERIAL

Preoperative ultrasound before sentinel lymph node biopsy in melanoma in the era of neoadjuvant treatment: a meta-analysis of diagnostic performance and cost analysis

Contents

A. Full search strategy systematic review 3

B. Assumptions of costs and chances 4

B.1 Assumptions of costs 4

B.2 Assumptions of chances 5

C. Full quality assessments 6

D. Diagnostic performance and predictive values of preoperative ultrasound and ultrasound-guided fine-needle aspiration cytology stratified by T Stage 7

E. Scenario analyses 8

F. Association between Breslow thickness and sensitivity of ultrasound / ultrasound-guided fine-needle aspiration in included studies 10

References 11

A. Full search strategy systematic review

Database searched	Platform	Years of coverage	Records	Records after duplicates removed
**Medline ALL**	Ovid	1946 - Present	463	460
**Embase**	Embase.com	1971 - Present	1063	667
**Web of Science Core Collection***	Web of Knowledge	1975 - Present	565	188
**Total**			**2091**	**1315**

*Science Citation Index Expanded (1975-present) ; Social Sciences Citation Index (1975-present) ; Arts & Humanities Citation Index (1975-present) ; Conference Proceedings Citation Index- Science (1990-present) ; Conference Proceedings Citation Index- Social Science & Humanities (1990-present) ; Emerging Sources Citation Index (2005-present)

No other database limits were used than those specified in the search strategies

medline

(exp Melanoma / OR (melanoma* OR melanocarcinoma* OR melanomalignoma*).ab,ti,kw.) AND (exp Ultrasonography / OR (echogra* OR ultrasound* OR Ultrasonogra* OR sonogra*).ab,ti,kw. OR (us).ti.) AND (Sentinel Lymph Node / OR Sentinel Lymph Node Biopsy / OR Lymph Node Excision / OR Lymphatic Metastasis / OR ((sentinel* ADJ3 node*) OR lymph-node* OR lymphogra*).ab,ti,kw. OR (sln).ti.) NOT (exp animals/ NOT humans/) AND english.la.

embase

(melanoma/exp OR (melanoma* OR melanocarcinoma* OR melanomalignoma*):ab,ti,kw) AND (echography/exp OR (echogra* OR ultrasound* OR Ultrasonogra* OR sonogra*):Ab,ti,kw OR (us):ti) AND ('sentinel lymph node'/de OR 'sentinel lymph node biopsy'/de OR 'lymph node dissection'/de OR 'lymph node metastasis'/de OR ((sentinel* NEAR/3 node*) OR lymph-node* OR lymphogra*):ab,ti,kw OR (sln):ti) NOT [conference abstract]/lim NOT ([animals]/lim NOT [humans]/lim) AND [english]/lim

Web of science

TS=((melanoma* OR melanocarcinoma* OR melanomalignoma*)) AND (TS=(echogra* OR ultrasound* OR Ultrasonogra* OR sonogra*) OR TI=(us)) AND (TS=((sentinel* NEAR/2 node*) OR lymph-node* OR lymphogra*) OR TI=(sln)) AND LA=(English) NOT DT=(Meeting Abstract OR Meeting Summary)

B. Assumptions of costs and chances

B.1 Assumptions of costs

Adjuvant Immunotherapy	*Nivolumab*[1]	*Pembrolizumab*[1]	*Dabrafenib/ trametinib*[1]	*Nivolumab following ipilimumab-nivolumab*[1]	*Dabrafenib/ trametinib following ipilimumab-nivolumab*[1]	*Pembrolizumab following neoadjuvant pembrolizumab*[1]
Dose	240mg, 15 cycles	200mg 18 cycles	dabrafenib 150 mg twice daily trametinib 2 mg once daily for 52 weeks	240mg, 11 cycles	dabrafenib 150 mg twice daily trametinib 2 mg once daily for 46 weeks	200mg 15 cycles
Drug acquisition costs	€63,183·90	€94,477·64	€144,859·03	€53,463·30	€128,133·46	€78,731·40
Drug administration costs	€4,614·99	€2,831·47	€12·51	€3,904·99	€12·51	€2,359·55
Total	€67,798·89	€97,309·15	€144,859·03	€57,368·29	€128,145·97	€81,090·95

Neoadjuvant immunotherapy	***Nivolumab/ ipilimumab***[1]	***Pembrolizumab***[1]
Dose	Nivolumab 240mg, ipilimumab 80mg, 2 cycles	200 mg, 3 cycles
Drug acquisition costs	€14,665·77	€15,746·28
Drug administration costs	€710·00	€471·91
Total	€15,375·77	€16.218·19

Other costs	
Ultrasound[2]	€121·53
FNAC[3]	€209·50
SLNB[3]	€1,907·70
IND*	€1,907·70
Follow up stage II[2,4]	€1,427·50
Follow up stage III[2,5]	€2,769·35

* Index node procedure costs assumed equivalent to SLNB.

Abbreviations: FNAC, fine-needle aspiration cytology; SLNB, sentinel lymph node biopsy; IND, index node dissection

B.2 Assumptions of chances

	Percentages
**Response after nivolumab plus ipilimumab**[6]	
MPR	59%
Non-MPR	41%
**BRAF mutation**[7]	
Present	46%
Absent	54%
**BRAF mutation after neoadjuvant therapy**[6]	
Present	63%
Absent	37%
**T1b**	
SN positivity	5·2%
Positive SN with a tumor burden >1mm[8]	28%
**T2a**	
SN positivity	10·7%
Positive SN with a tumor burden >1mm[8]	37%
**T2b**	14·5%
**T3**	24·7%
**T4**	34·4%
**>T2b**[8]	25·5%

Abbreviations: MPR, major pathological response, SN, sentinel node

C. Full quality assessments

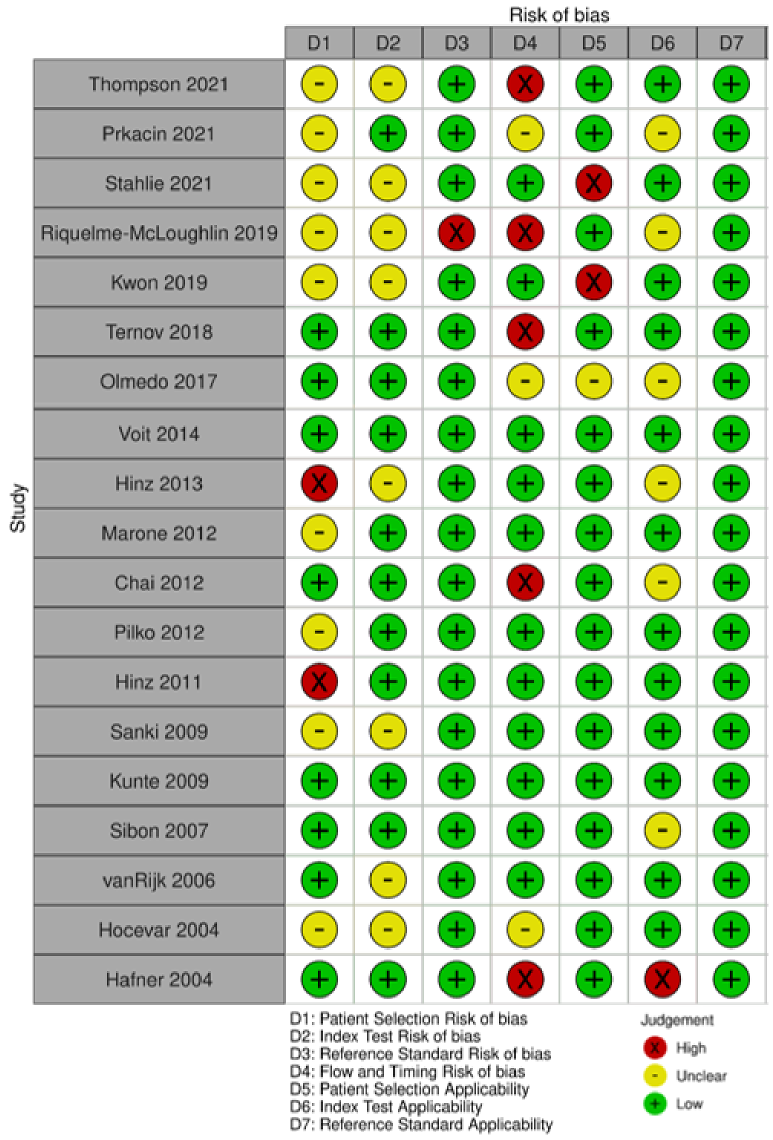

Figure C1: Traffic light plot

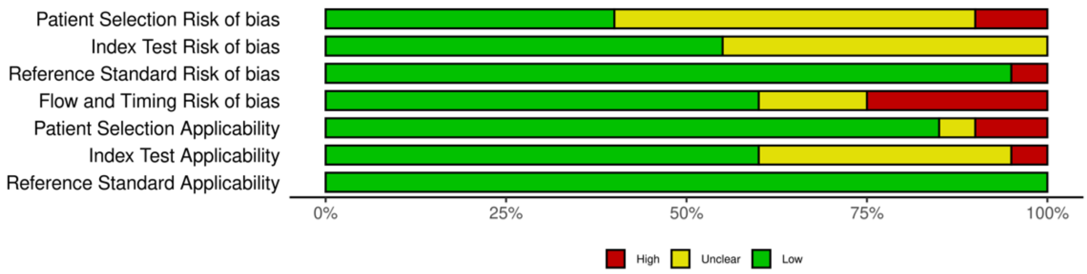

Figure C2: Summary plot

D. Diagnostic performance and predictive values of preoperative ultrasound and ultrasound-guided fine-needle aspiration cytology stratified by T Stage

T stage	SN+	PPV (ultrasound)	NPV (ultrasound)	Expected ultrasound positivity	PPV (ultrasound-FNAC)	NPV (ultrasound-FNAC)	Expected ultrasound-FNAC positivity
T1b	5·2%	19·5%	96·2%	9·0%	96·1%	98·2%	1·4%
T2a	10·7%	34·6%	92·1%	10·4%	98·2%	96·2%	2·7%
T2b	14·5%	42·8%	89·1%	11·4%	98·7%	94·7%	3·7%
T3	24·7%	59·2%	80·9%	14·0%	99·3%	90·2%	6·1%
T4	34·4%	69·9%	72·6%	16·5%	99·6%	85·2%	8·5%
≥T2b	25·5%	60·2%	80·3%	14·2%	99·4%	89·8%	6·3%

Abbreviations: SN, sentinel node; PPV, positive predictive value; NPV, negative predictive value

E. Scenario analyses

T1b: Cost analysis comparing primary SLNB versus preoperative ultrasound-FNAC for T1b melanoma, including scenarios for adjuvant treatment following a positive sentinel node and for patients receiving neoadjuvant ipilimumab–nivolumab with non-MPR in the index node resection.

Adjuvant following a positive SN	Adjuvant following non-MPR after neoadjuvant ipilimumab-nivolumab	Primary SLNB	Preoperative ultrasound-FNAC	Difference
Nivolumab	Nivolumab	€4,341·89	€4,847·07	-€505·18
Pembrolizumab	Nivolumab	€4,771·56	€5,143·05	-€371·49
Nivolumab	Nivolumab/dabrafenib-trametinib	€4,341·89	€5,154·71	-€812·82
Pembrolizumab	Nivolumab/dabrafenib-trametinib	€4,774·56	€5,450·69	-€679·13
Nivolumab/dabrafenib-trametinib	Nivolumab/dabrafenib-trametinib	€4,858·01	€5,473·43	-€615·42
Pembrolizumab/dabrafenib-trametinib	Pembrolizumab/dabrafenib-trametinib	€5,090·03	€5,670·07	-€580·05

Abbreviations: SN, sentinel node; MPR, major pathological response, SLNB, sentinel lymph node biopsy; ultrasound; FNAC, fine-needle aspiration cytology

T2a: Cost analysis comparing primary SLNB versus preoperative ultrasound-FNAC for T2a melanoma, including scenarios for adjuvant treatment following a positive sentinel node and for patients receiving neoadjuvant ipilimumab–nivolumab with non-MPR in the index node resection.

Adjuvant following a positive SN	Adjuvant following non-MPR after neoadjuvant ipilimumab-nivolumab	Primary SLNB	Preoperative ultrasound-FNAC	Difference
Nivolumab	Nivolumab	€6,072·48	€6,729·28	-€656·79
Pembrolizumab	Nivolumab	€7,240·79	€7,534·08	-€293·29
Nivolumab	Nivolumab/dabrafenib-trametinib	€6,072·48	€7,349·08	-€1,276·59
Pembrolizumab	Nivolumab/dabrafenib-trametinib	€7240·79	€8,153·88	-€913·09
Nivolumab/dabrafenib-trametinib	Nivolumab/dabrafenib-trametinib	€7,475·86	€8,249·52	-€773·67
Pembrolizumab/dabrafenib-trametinib	Pembrolizumab/dabrafenib-trametinib	€8,106·74	€8,750·41	-€643·66

Abbreviations: SN, sentinel node; MPR, major pathological response, SLNB, sentinel lymph node biopsy; FNAC, fine-needle aspiration cytology

Neoadjuvant pembrolizumab with adjuvant nivolumab

Adjuvant	Neoadjuvant	Primary SLNB	Preoperative ultrasound-FNAC	Difference
Nivolumab	Pembrolizumab	€20,966·09	€22,932·90	-€1,966·82
Nivolumab	MPR based (10%)	€20,966·09	€22,376·06	-€1,409·97
Nivolumab	MPR based (20%)	€20,966·09	€21,728·56	-€762·47
Nivolumab	MPR based (25%)	€20,966·09	€21,404·80	-€438·72
Nivolumab	MPR based (30%)	€20,966·09	€21,081·05	-€114·96
Nivolumab	MPR based (35%)	€20,966·09	€20,757·30	€208·79
Nivolumab	MPR based (40%)	€20,966·09	€20,433·55	€532·54
Nivolumab	MPR based (45%)	€20,966·09	€20,109·79	€856·29
Nivolumab	MPR based (50%)	€20,966·09	€19,786·04	€1,180·05

Abbreviations: MPR, major pathological response, SLNB, sentinel lymph node biopsy; FNAC, fine-needle aspiration cytology

Neoadjuvant pembrolizumab with adjuvant pembrolizumab

Adjuvant	Neoadjuvant	SLNB	Ultrasound	Difference
Pembrolizumab	Pembrolizumab	€28,491·20	€28,693·81	-€202·61
Pembrolizumab	MPR based (10%)	€28,491·20	€28,046·31	€444·90
Pembrolizumab	MPR based (20%)	€28,491·20	€27,398·80	€1,092·40
Pembrolizumab	MPR based (25%)	€28,491·20	€27,075·05	€1,416·15
Pembrolizumab	MPR based (30%)	€28,491·20	€26,751·30	€1,739·91
Pembrolizumab	MPR based (35%)	€28,491·20	€26,427·55	€2,063·66
Pembrolizumab	MPR based (40%)	€28,491·20	€26,103·79	€2,387·41
Pembrolizumab	MPR based (45%)	€28,491·20	€25,780·04	€2,711·16
Pembrolizumab	MPR based (50%)	€28,491·20	€25,456·29	€3,034·92

Abbreviations: MPR, major pathological response, SLNB, sentinel lymph node biopsy

F. Association between Breslow thickness and sensitivity of ultrasound / ultrasound-guided fine-needle aspiration in included studies

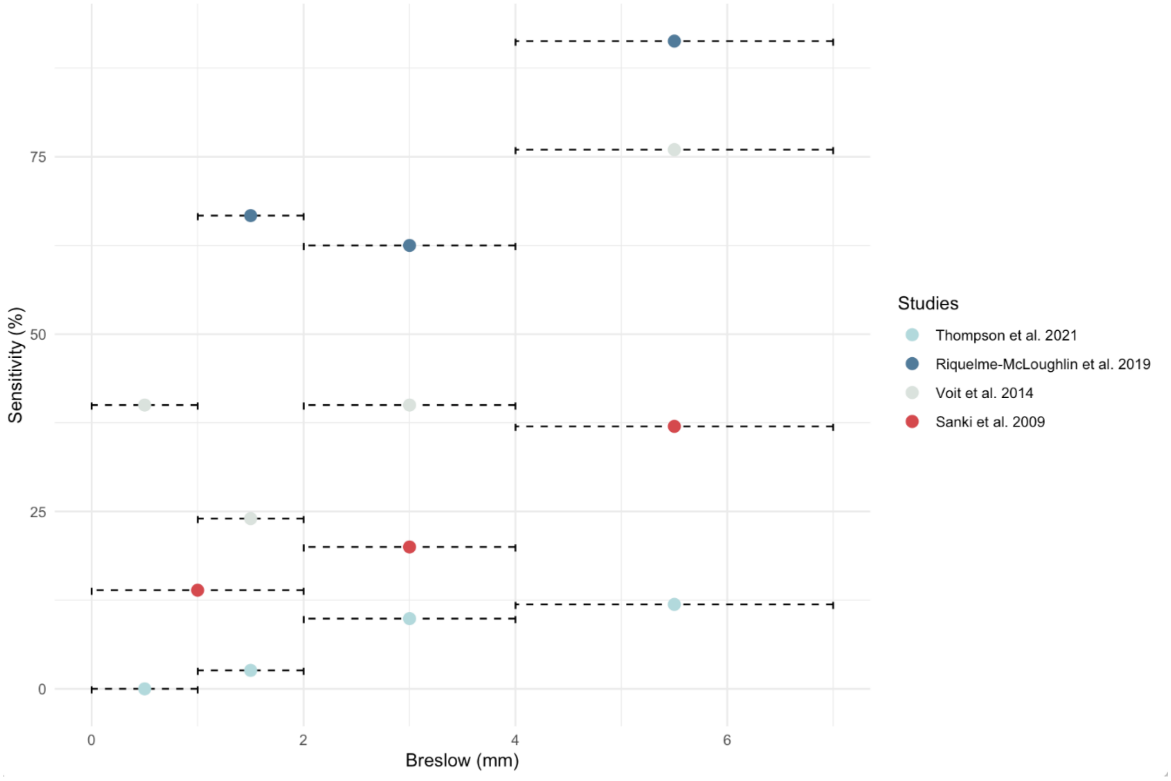

Figure G: Association of sensitivity and Breslow thickness

References

1. Zorginstituut Nederland. Medicijnkosten.nl. 10-06-2025. https://www.medicijnkosten.nl/ (accessed 16-06-2025.
2. Hakkaart-van Roijen LPS, Peeters S, Kanters T. Kostenhandleiding voor Economische Evaluaties in de Gezondheidszorg: Methodologie en Referentieprijzen Herziene Versie 2024: Zorginstituut Nederland, 2024.
3. Nederlandse Zorgautoriteit. Over de Nederlandse Zorgautoriteit. https://www.nza.nl/over-de-nza (accessed August 14, 2025.
4. Nederlandse Internisten Vereniging, Nederlandse Vereniging voor Heelkunde. Follow-up bij melanoom stadium IB en II. 2024. https://richtlijnendatabase.nl/richtlijn/melanoom/follow-up_-_melanoom/follow-up_bij_melanoom_stadium_ib_en_ii.html (accessed August 14, 2025.
5. Nederlandse Internisten Vereniging, Nederlandse Vereniging voor Heelkunde. Follow-up bij locoregionaal gemetastaseerd melanoom (stadium III) 2024. https://richtlijnendatabase.nl/richtlijn/melanoom/follow-up_-_melanoom/follow-up_bij_locoregionaal_gemetastaseerd_melanoom_stadium_iii.html (accessed August 14, 2025.
6. Blank CU, Lucas MW, Scolyer RA, et al. Neoadjuvant Nivolumab and Ipilimumab in Resectable Stage III Melanoma. *N Engl J Med* 2024; **391**(18): 1696-708.
7. Barbour AP, Tang YH, Armour N, et al. BRAF mutation status is an independent prognostic factor for resected stage IIIB and IIIC melanoma: implications for melanoma staging and adjuvant therapy. *Eur J Cancer* 2014; **50**(15): 2668-76.
8. Hafner J, Schmid MH, Kempf W, et al. Baseline staging in cutaneous malignant melanoma. *Br J Dermatol* 2004; **150**(4): 677-86.
